# EXPLORING DIET’S IMPACT ON CARDIOMETABOLIC AND COGNITIVE HEALTH IN AMERICAN INDIANS

**DOI:** 10.1093/geroni/igae098.3971

**Published:** 2024-12-31

**Authors:** Marianne Chanti-Ketterl, Jennifer Jones-Locklear, Jada Brooks, Cierra Williams, Jessica Locklear, Leslie Strickland-Yanick, Heather Whitson

**Affiliations:** Duke University, Durham, North Carolina, United States; University of North Carolina Pembroke, North Carolina, United States; university of North Carolina, Chapel Hill, North Carolina, United States; Duke University, Durham, North Carolina, United States; University of North Carolina Pembroke, North Carolina, United States; University of North Carolina Pembroke, North Carolina, United States; Duke University, Durham, North Carolina, United States

## Abstract

**Objective:**

This study explores the link between cardiometabolic biomarkers and neurocognition in older American Indians, focusing on diet quality as measured by the Healthy Eating Index (HEI).

**Methods:**

Participants provided biological specimens and completed health, diet, and exposome questionnaires in a mobile clinic. Cardiometabolic biomarkers (lipids, glucose) were measured with a Cholestech LDX Analyzer and log-transformed. Neurocognition was assessed using the Montreal Cognitive Assessment (MoCA). OLS regressions, adjusted for age and education, examined the association between biomarkers and neurocognition across HEI tertiles to assess effect modification.

**Results:**

Across three months, we enrolled 41 participants, predominantly women (76%), with a mean age of 72 years (SD 7.8) and an average of 12.6 years of education (SD 3.2). HDL-Cholesterol levels were higher in women than in men (n=39, p=0.008). The mean HEI score was 64.3(SD 11.4), with varied diet quality among participants; lowest HEI tertile (44.2-58.1), highest HEI tertile (71.4-84). In the lowest HEI-tertile, higher MoCA scores were associated with higher LDL-C, lower triglycerides, and higher glucose. In contrast, the mid-HEI-tertile showed higher MoCA scores associated with lower TC and lower glucose, but higher LDL-C and triglycerides. For the highest HEI-tertile, higher MoCA scores were associated only with lower triglycerides.

**Discussion:**

These preliminary findings suggest the relationship between cardiometabolic markers and cognitive function may depend on diet quality. Understanding the role of nutrition on brain and cardiometabolic health among older American Indians may lead to culturally tailored nutritional interventions to promote brain health in aging. Further analysis of specific nutrients, the microbiome, and the metabolome are forthcoming.

